# Wire-assisted modification of Havst catheter stripping of the great saphenous vein trunk

**DOI:** 10.3389/fsurg.2025.1691757

**Published:** 2025-11-27

**Authors:** Xiaowen Lin, Pengfei Li, Lijuan Zhuang

**Affiliations:** 1 Department of Vascular Surgery, Fujian University of Traditional Chinese Medicine Affiliated People's Hospital, Fuzhou, Fujian, China; 2 Fujian Maternity and Child Health Hospital, Fuzhou, Fujian, China

**Keywords:** great saphenous varicose veins, Havst catheter, wire-assisted, minimally invasive surgery, tumescent anesthesia

## Abstract

**Objective:**

To evaluate the therapeutic outcomes using Havst catheter stripping vs. a wire-assisted modification of Havst catheter stripping the trunk of great saphenous vein (GSV) above knee.

**Methods:**

Data were collected from 120 patients who underwent stripping of the GSV trunk using a Havst catheter from January 2025 to June 2025; 60 patients underwent the conventional approach to Havst catheter stripping and 60 patients underwent a wire-assisted modification of Havst catheter stripping. Age, gender, Clinical class, Stripping time of the GSV trunk (including tumescent anesthesia), length of the stripped GSV trunk above knee, intraoperative Visual Analogue Scale (VAS) pain score, and success of surgery were compared between treatment groups.

**Results:**

A wire-assisted modification of Havst catheter stripping showed significant advantages over conventional approach of Havst catheter in terms of longer median (interquartile range, IQR) Stripping length of GSV trunk 384 (361, 407) vs. 321 (309, 334) cm, *p* < 0.0001, and higher success rate (96.67% vs. 76.67%, *p* = 0.0031), and shorter median (interquartile range, IQR) thigh incision length 11 (10, 12) vs. 12 (11, 14) cm, *p* = 0.0006). There were no significant differences in age (*t*-tests, mean ± standard deviation) (55.23 ± 10.00 vs. 54.60 ± 11.12 years, *p* = 0.7435), gender (*p* = 0.0956), Clinical class (*p* = 0.4395), Stripping time (*t*-tests, mean ± standard deviation) (410 ± 18 vs. 407 ± 17 s, *p* = 0.3816), intraoperative VAS pain score median (interquartile range, IQR) [(1 (1, 2) vs. 1 (1, 2), *p* = 0.8897].

**Conclusion:**

The wire-assisted modification of Havst catheter stripping enabled faster and the longer segment of the GSV trunk above knee was stripped compared to the conventional approach to Havst catheter stripping.

## 1 Introduction

**Figure 1:**
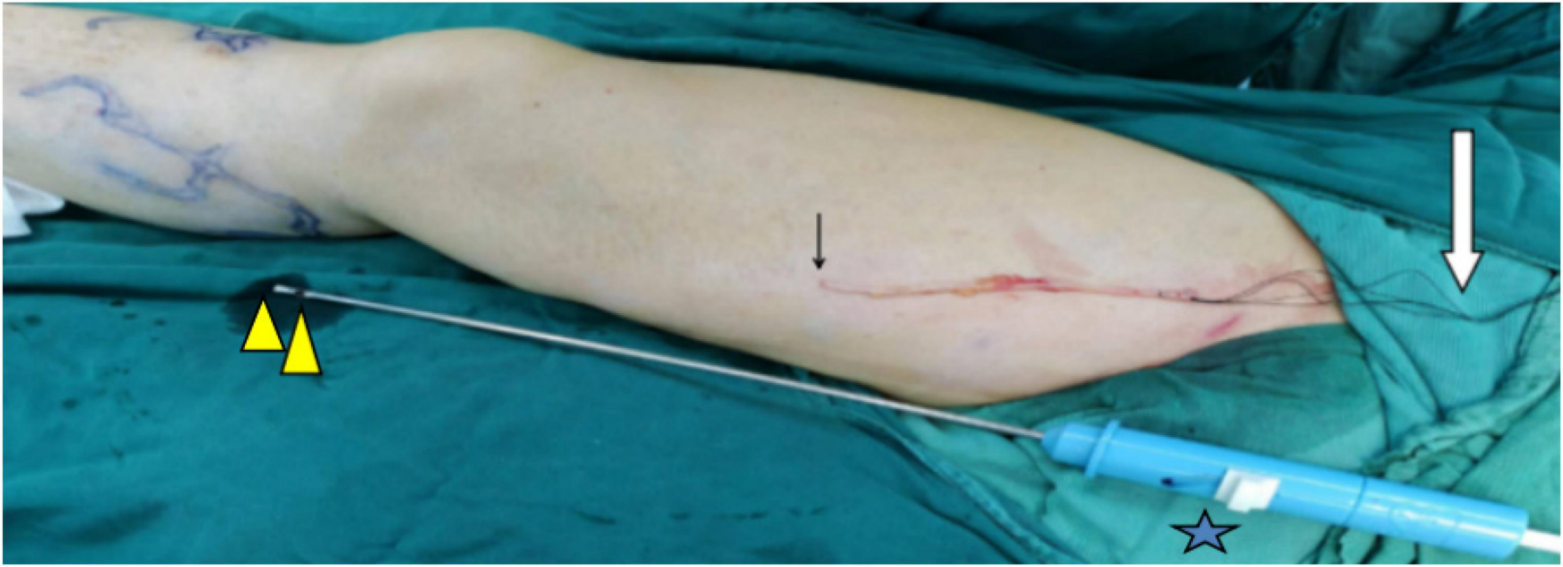
Conventional approach to Havst catheter stripping. Small arrow: There is an unplanned disconnection. Big arrow: 4 # silk thread used for traction. The tip and side holes of the stripping catheter are indicated by the triangles. The blade button is marked with a pentagram.

**Figure 2:**
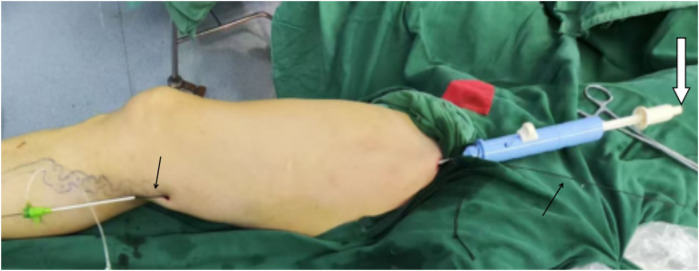
Wire-assisted modification of Havst catheter stripping. Small arrow: Stretch the 4 # guide wire to guide and support the downward movement of the catheter. Big arrow: Inject swelling fluid through the tail tube to establish a swelling fluid tunnel around the main trunk of the great saphenous vein.

With the advancement of healthcare and emphasis on speed of patient recovery, minimally invasive techniques have emerged as the primary treatment approach for large varicose veins (1). In China, a 2018 survey indicated that 68.3% (2) of patients with varicose veins underwent traditional stripping of the GSV trunk. In recent years, a stripping catheter for the great saphenous vein trunk in the thigh region has been introduced in China and adopted in clinical procedures. The Havst catheter (Hangzhou Tiancheng Pharmaceutical Co., Ltd., China) (Patent No: ZL2007 1 0194557.3) is a vein stripping catheter designed for minimally invasive ablation of the great saphenous vein trunk in the thigh area (For details of the instrument's structure, see Figures 1, 2). A key feature of this catheter is its ability to perform tumescent anesthesia by injecting tumescent fluid through the terminal hole, followed by stripping along the adventitia of the great saphenous vein trunk. It enables complete stripping of the thigh segment of the great saphenous vein through a single incision. However, several challenges are commonly encountered when stripping the GSV. The Havst catheter must be advanced along the GSV trunk, often relying on surgeon experience or intuition for guidance, without directional support. Advancing the Havst catheter may be difficult due to an improper angle, insufficient tumescent solution, the presence of branches, or resistance. Repeated angulation or use of excessive force may damage the vein trunk, resulting in vein transection during the procedure.

By pre-positioning a 0.035 inch introducer wire in the GSV trunk, the Havst catheter can be advanced along the wire, providing directional guidance and support and more minimally invasive stripping. The guidewire-assisted technique has been granted a Patent (Patent No. ZL 2024 2 1767804.X, Inventor: Xiaowen Lin). The objective of this study was to evaluate the therapeutic outcomes of the conventional approach to Havst catheter stripping vs. a wire-assisted modification of Havst catheter stripping of the GSV trunk above knee.

## 2 Materials and methods

### 2.1 Patient population

Patients who underwent surgery for primary lower extremity varicosity at our hospital from January 2025 to June 2025 were eligible for this study. Inclusion criteria were: (1) Clinical class classified according to CEAP (Clinical-Etiology-Anatomy-Pathophysiology) between C2 and C6; (2) non severe tortuosity course of the GSV trunk; (3) informed consent obtained from the patient. Exclusion criteria were: (1) presence of deep venous abnormality in the lower extremities, such as iliac vein compression or a history of deep vein thrombosis; (2) clinical class <C2; (3) presence of lower extremity artery occlusive disease; (4) poor general condition or comorbidities that contraindicated surgical intervention.

The guidewire-assisted stripping trunk of the GSV is an innovative technique. We submitted an application to the hospital for the implementation of this new technique (Ethical approval number: 2024-087-01), which was approved by the hospital administration. This study collected relevant data from patients with GSV disease who underwent treatment from January 01, 2025 to June 30, 2025. A comparative study of enrollment between the two patient groups. Preoperative color Doppler ultrasound was performed to evaluate the saphenous vein trunk in the thigh segment. Color Doppler ultrasound should evaluate the course, diameter (3), and skin-to-vein depth of the great saphenous vein (GSV) trunk, saphenofemoral junction (SFJ) valve reflux status, as well as the number, course, diameter of tributaries, and the presence of communicating and perforating veins. Eligible patients were consecutively numbered from 1 to 10 in chronological order of their surgical procedures, with odd-numbered patients allocated to the Havst group and even-numbered patients to the wire-assisted modified stripping group. A total of 120 patients were enrolled, with 60 cases assigned to the Havst group and 60 cases to the modified stripping group.

### 2.2 Procedures

#### 2.2.1 Preoperative preparation

Upon admission, patients underwent relevant preoperative examinations and tests to exclude any surgical contraindications. The contour of the superficial varicose veins of the affected limb was marked while the patients stood, identifying prominent areas. Surgery was performed under tumescent anesthesia. A tumescent solution was prepared using 20 mL of 1% ropivacaine, 200 mg of 2% lidocaine, 0.25 mg of 0.1% epinephrine, and 500 mL of normal saline.

#### 2.2.2 Management trunk of the GSV above knee

Patients were placed in the supine position, followed by disinfection and draping. Tumescent anesthesia was administered in the inguinal surgical area. Under ultrasound guidance, a skin incision was made approximately 2 cm distal to the entrance of the saphenofemoral vein, below the inguinal ligament. The GSV trunk was grasped with curved forceps, and surrounding tissue was dissected to expose the vein. Proximal ligation was performed.

For the conventional approach to Havst catheter stripping, the cephalad aspect of the GSV root incision in the inguinal region was ligated with 7–0 silk suture. The caudal of the GSV incision was ligated with 4–0 silk suture, leaving the suture untrimmed. The 4–0 silk suture at the groin incision was threaded through the tip of the Havst catheter, the silk suture was exteriorized through the side port of the catheter, the GSV was exteriorized through the catheter's side port by traction on the silk suture. Adequate tumescent anesthesia solution was infused via the catheter's distal port to establish a perivenous fluid cushion. The catheter was sequentially advanced along the above-knee GSV trunk from the groin region to the knee joint under ultrasound guidance. The cutting button was activated to transect the GSV trunk. The technical success of the surgery was defined by the complete stripping of the GSV trunk above knee below the level of the upper edge of the patella using the stripping catheter (Figure 1).

For the wire-assisted modification of Havst catheter stripping, under ultrasound guidance, the GSV was punctured at the knee, and a 6F vascular sheath (Teleflex, Japan) was inserted. A 0.035 inch introducer wire was advanced through the sheath, and its tip was directed through the distal end of the vein incision. The proximal stump was routinely ligated, while the distal stump was secured with two 4–0 silk ligatures in the inguinal region. The distal GSV was then cannulated into the Havst stripping catheter, with the traction sutures threaded through the catheter lumen and exteriorized through its side port. The operator maintained cephalad traction on both the guidewire and traction sutures with one hand, while simultaneously advancing the catheter toward the foot end with the other hand. Concurrently, the assistant applied caudal traction on the guidewire to establish a supportive rail (“support wire technique”) for facilitated catheter progression. Under continuous traction of the guide sutures and wire, the Havst catheter was advanced along the adventitial plane of the GSV trunk to perform circumferential stripping. The procedure was terminated at the knee level, where the GSV was transected. During catheter advancement, tumescent solution was injected through the catheter until the catheter tip reached the puncture point of the sheath. The introducer wire was then removed, and the GSV trunk was transected. Take out the stripped GSV. The total procedure time (including tumescent anesthesia administration) was recorded. The length of the stripped GSV above knee was measured (Figure 2).

In the event of an unintended vein transection during the conventional approach to Havst catheter stripping, the distal segment of the GSV trunk was removed, under ultrasound guidance, and the residual vein was dissected. When using solely the Havst catheter for GSV trunk stripping, an unplanned transection occurred, necessitating additional thigh incision. Under color Doppler ultrasound guidance, the residual GSV trunk required complete surgical removal to prevent neovascularization.

#### 2.2.3 Intraoperative pain assessment

Intraoperative Visual Analog Scale (VAS) pain scores were assessed during great saphenous vein (GSV) trunk stripping. Pain was assessed using the Visual Analog Scale (VAS) at 24 h after surgery (0–10 scale: 0–3 indicating mild pain that is tolerable, 4–6 indicating moderate pain that affects sleep but is still tolerable, 7–10 indicating severe pain that impacts appetite and sleep).

#### 2.2.4 Cosmetic suturing of the inguinal incision

A cosmetic suture was used to close the inguinal incision. A gauze pad was applied to exert localized pressure along the course of the GSV trunk and its branches, followed by application of an elastic compression bandage with appropriate pressure.

#### 2.2.5 Postoperative management

All patients received oral rivaroxaban 10 mg daily for anticoagulation within the first 5 postoperative days. During the hospitalization period, pneumatic compression therapy was used to promote venous return, and patients were encouraged to ambulate.

### 2.3 Statistical methods

Statistical analysis was performed using SPSS version 22.0. For the analysis of measurement data, it is necessary to determine whether it follows a normal distribution. If the data is normally distributed, parametric tests (such as *t*-tests and ANOVA) should be prioritized, and the results should be described using mean ± standard deviation. If the data does not follow a normal distribution, non-parametric tests (such as the Mann–Whitney *U*-test) should be adopted, and the results should be described using median (interquartile range). Categorical data were compared using the Chi-square (*χ*^2^) test. Ordinal data were compared using the rank-sum test. A *p*-value of <0.05 was considered statistically significant.

## 3 Results

**Figure 3:**
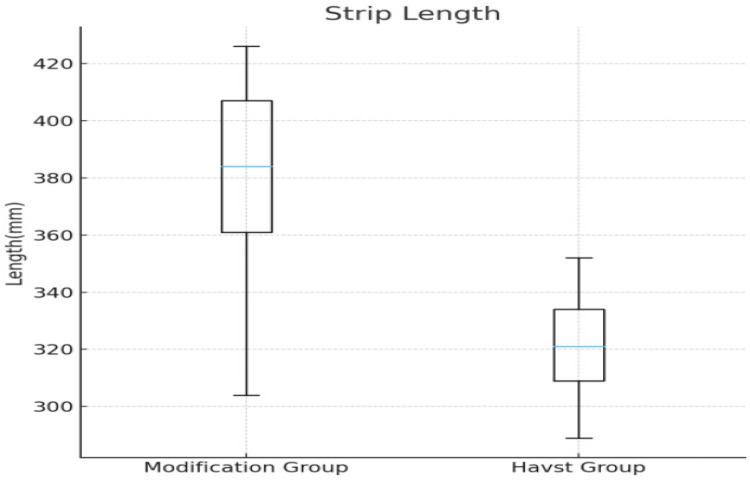
Comparison of the stripping length of the main trunk of the great saphenous vein in the thigh between two groups.

**Figure 4:**
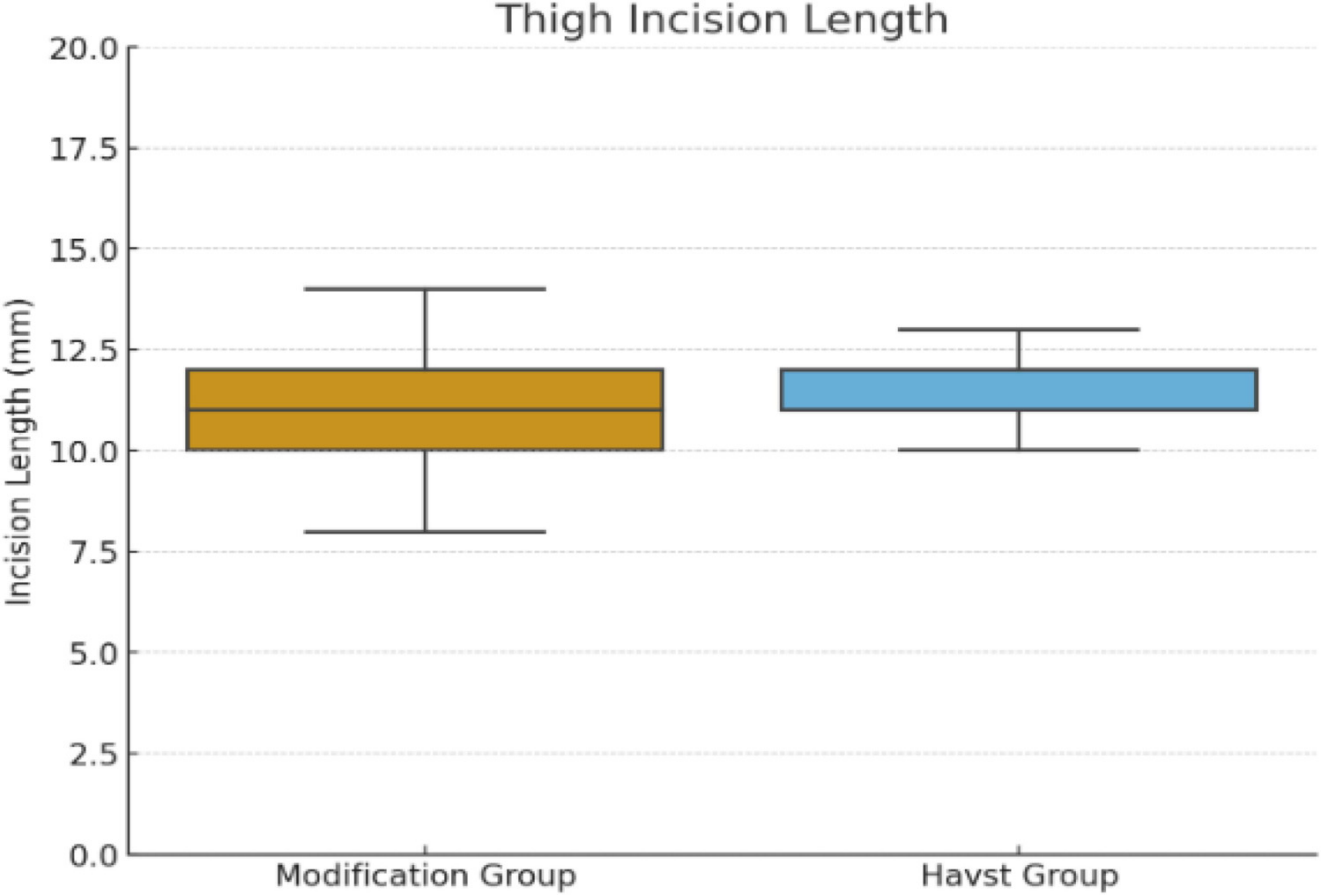
Comparison of the incision length of the main trunk of the great saphenous vein in the thigh between two groups.

**Figure 5:**
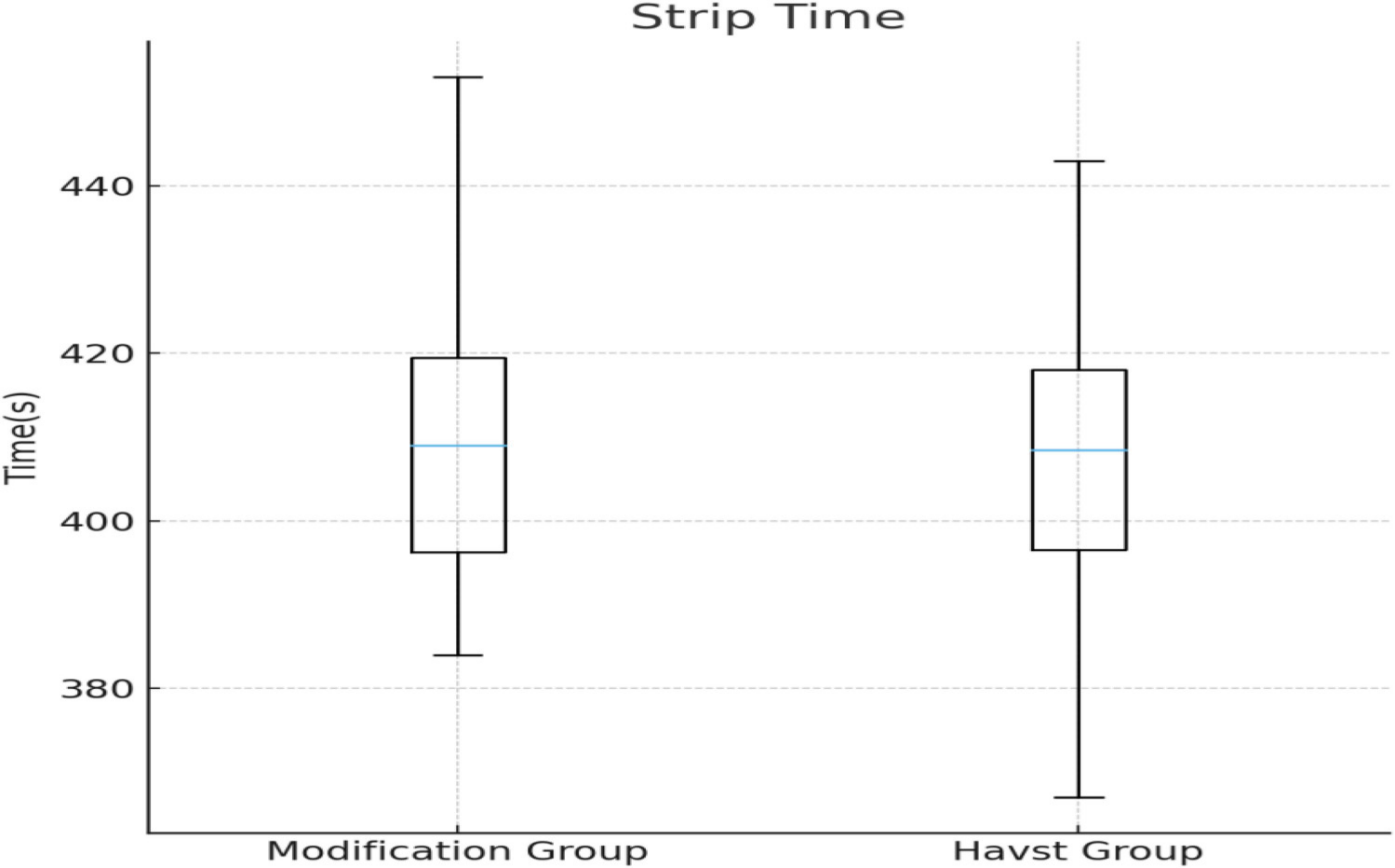
Comparison of the stripping time of the main trunk of the great saphenous vein in the thigh between two groups.

**Figure 6:**
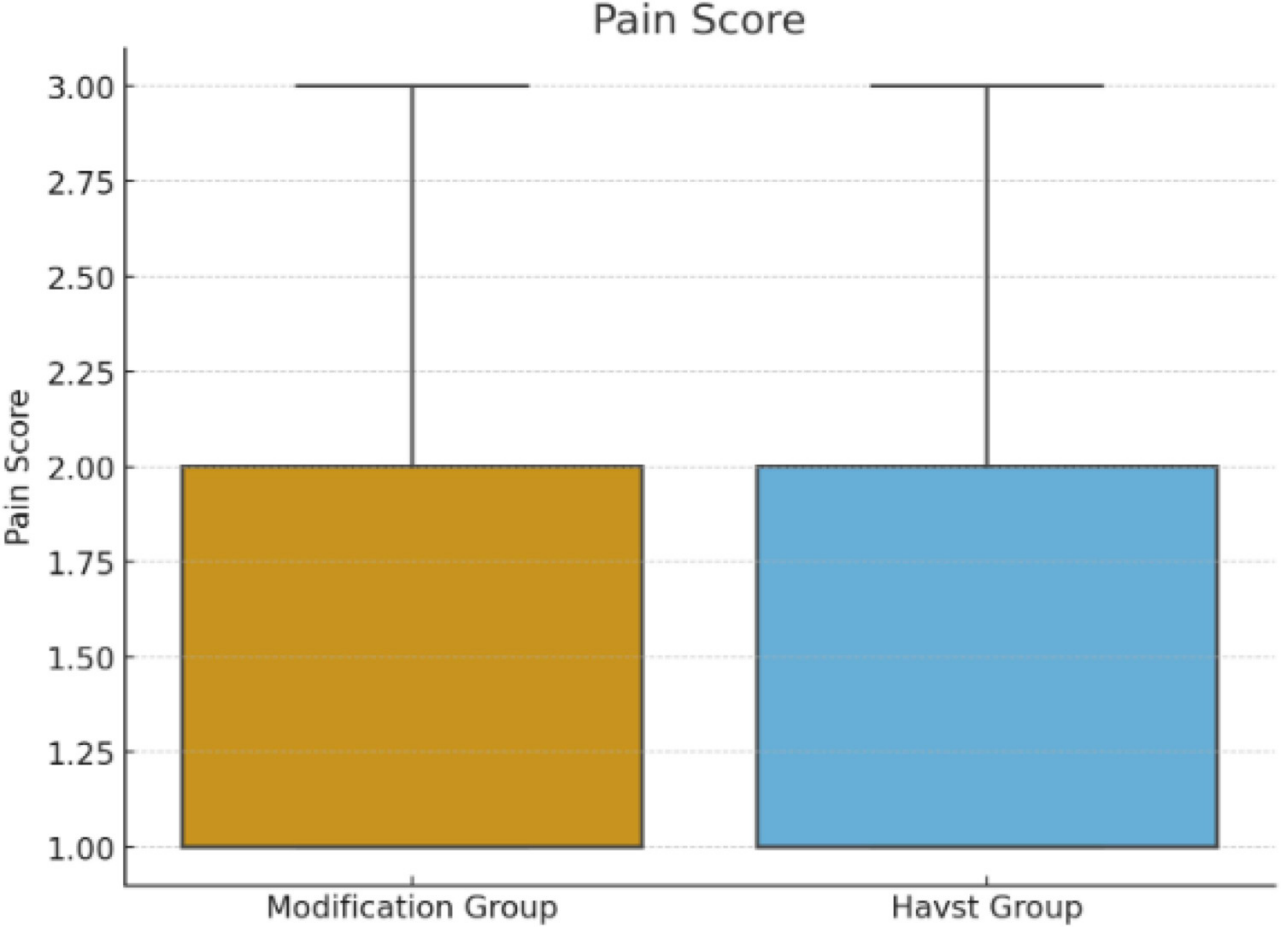
Comparison of the pain score of the main trunk of the great saphenous vein in the thigh between two groups.

A wire-assisted modification of Havst catheter stripping showed significant advantages over conventional approach of Havst catheter in terms of longer median (interquartile range, IQR) Stripping length of GSV trunk [(384 (361, 407) vs. 321 (309, 334) cm, *p* < 0.0001] (Figure 3), and higher success rate (96.67% vs. 76.67%, *p* = 0.0031), and shorter median (interquartile range, IQR) thigh incision length 11 (10, 12) vs. 12 (11, 14) cm, *p* = 0.0006 (Figure 4). There were no significant differences in age (*t*-tests, mean ± standard deviation) (55.23 ± 10.00 vs. 54.60 ± 11.12 years, *p* = 0.7435), gender (*p* = 0.0956), Clinical class (*p* = 0.4395), Stripping time (*t*-tests, mean ± standard deviation) (410 ± 18 vs. 407 ± 17 s, *p* = 0.3816) (Figure 5), intraoperative VAS pain score median (interquartile range, IQR) [1 (1, 2) vs. 1 (1, 2), *p* = 0.8897] (Figure 6, Table 1).

**Table 1:** Demographic and clinical data.

Variable	Modified stripping	Havst stripping	Statistic	*P*-value
Demographic data				
Male	30	20	*χ*² = 2.777	0.0956
Female	30	40		
Age (years)	55.23 ± 10.00	54.60 ± 11.12		0.7435
Clinical grade				
C2	10	16	Z = −0.743	0.4395
C3	20	16		
C4	22	21		
C5	5	3		
C6	3	4		
Clinical outcomes				
thigh incision length (cm)	11 (10, 12)	12 (11, 14)	U = 2,451.000	0.0006*
Length of the stripped GSV trunk (cm)	384 (361, 407)	321 (309, 334)	U = 308.500	0.0000*
Stripping time of the GSV trunk (s)	410 ± 18	407 ± 17	t = −0.878	0.3816
24 h VAS pain score	1 (1, 2)	1 (1, 2)	U = 1,775.500	0.8897
Success of surgery				
Success	58	46	*χ*^2^ = 8.726	0.0031*
Failure	2	14		

Gender is a categorical variable and was compared with the chi-square test.

Clinical classification was ordinal data, which were compared with the rank-sum test.

Success rate was measured as counts and was compared with the chi-square test. An asterisk (*) indicates a statistically significant difference between the two groups (*P* < 0.05).

## 4 Discussion

Endovascular thermal ablation has emerged as the treatment of choice for both physicians and patients owing to its minimally invasive nature. However, thermal ablation therapy is not suitable for all cases. For instance, cases with a great saphenous vein trunk diameter greater than 1.2 cm (4) or overly superficial location of the vein trunk may not be ideal candidates. In China, A questionnaire based article published in 2018 pointed out that: 68.3% of patients still undergo conventional stripping (2). Therefore, first of all, high ligation and stripping (HLS) retains its irreplaceable role in contemporary venous practice. Then, compared to traditional stripping procedures, this Havst catheter enables minimally invasive, rapid stripping of the GSV trunk under local anesthesia, with reduced postoperative pain and faster recovery. The significance of the Havst catheter-related research lies precisely in enabling a quick and easy minimally invasive stripping procedure. However, unplanned transection was observed during the stripping procedure in clinical practice. Reflecting on the causes of unplanned transection: it may be related to the stripping process using the Havst catheter. The stripping process lacks directional guidance and force support, leading to catheter tip-induced damage and transection of the GSV trunk. How to reduce unintended transection has become a critical challenge to overcome.

The versatile application of guidewires is commonly encountered in our clinical endovascular practice. Retrograde guidewire recanalization of lower extremity arterial occlusions (5). Treatment of coronary lesions using parallel wire technique (6) and buddy wire technique (7). In the treatment of thoracic aortic lesions, delivery of the Castor stent-graft (8) requires pre-establishment of a tension guidewire. This not only enables precise positioning and fixation, but also ensures stable passage through the stenotic segment. The guidewire provides continuous traction during the procedure to prevent vascular wall injury. Guidewire traction-assisted stent delivery through severely stenotic aortic lesions (9). Endovascular recanalization using a tension guidewire for the treatment of severe popliteal artery stenosis (10). These guidewires uniformly serve dual functions of guidance and support, enabling balloons or stents to traverse the lesion and reach the target vessel. Can guidewires be utilized to assist in great saphenous vein (GSV) stripping? And what type of guidewire would be most appropriate for this procedure?

Guidewires come in various types. They are categorized by diameter into: 0.014 inch, 0.018 inch, and 0.035 inch guidewires. Available lengths include: 150 cm and 250 cm. Functionally, they are classified as: guiding guidewires, superselective guidewires, and delivery guidewires. Based on stiffness, they are divided into: soft, stiff, and extra-stiff guidewires. Different guidewires possess distinct characteristics. By properly utilizing their unique features, we can achieve twice the result with half the effort. The 0.035 inch hydrophilic guidewire commonly used in interventions can meet the technical requirements for assisting Havst catheter in great saphenous vein stripping. With its low cost and high cost-effectiveness, it has become our preferred guidewire choice.

Guidewire-modified stripping demonstrates three clinical advantages. First, pre-placement of a 0.035 inch Glidewire throughout the GSV trunk lumen allows the Havst catheter stripping procedure to follow the guidewire's path. Traction on both ends of the guidewire provides directional guidance and mechanical support, facilitating faster stripping completion. A finding corroborated by the comparative stripping times between the two study groups. Second, guidewire traction ensures the stripping procedure follows the guidewire's path, reducing damage to surrounding tissues and making the stripping more minimally invasive. Third, the Havst catheter can strip along the guidewire to the vascular sheath entrance, enabling complete extraction of the entire GSV trunk and improving stripping success rates. This achieves longer segment removal and increasing the stripping success rate. The results from both groups demonstrate that the modified technique stripped longer segments of the GSV trunk and higher success rates, with statistically significant differences between the two groups.

Analysis of the outcomes from both groups demonstrated that the wire-assisted modified stripping technique significantly improved the success rate of complete vein stripping. The wire-assisted modified group demonstrated increased length of great saphenous vein (GSV) stripping and reduced surgical incision length in the thigh segment. To some extent, these three parameters demonstrate certain correlations. The technical success rate reflects complete stripping of the entire great saphenous vein (GSV) trunk in the thigh segment, with technical success defined by achieving a greater length of the stripped GSV and requiring no additional thigh incisions (resulting in shorter total incision length). Although the Havst catheter stripping group may cause more surrounding tissue damage, statistical results show no significant difference in VAS pain scores between the two groups. This may be attributed to the tumescent anesthetic solution injected along the GSV trunk through the catheter tip in both groups. The tumescent anesthesia solution not only reduces pain (11, 12), but also helps minimize bleeding, creates a hydroprotective cushion, and decreases trauma to surrounding tissues and nerves (13). Combined with femoral nerve block anesthesia, the procedure demonstrates enhanced therapeutic efficacy (14). Additionally, there was no significant difference in stripping time between the two groups. Although the wire-assisted technique provided mechanical support and directional guidance during stripping—potentially reducing the procedure duration—this advantage was offset by the additional time required for antegrade wire placement throughout the great saphenous vein (GSV) trunk.

In conclusion, this study showed that modifying the conventional approach to Havst catheter stripping of the GSV trunk by advancing the Havst catheter over a guidewire enhances the minimally invasive nature of the procedure and reduces complications, allowing more patients with lower extremity varicose veins to benefit from treatment. The mid- to long-term effectiveness of this technique requires further investigation.

## Data Availability

The raw data supporting the conclusions of this article will be made available by the authors, without undue reservation.
